# Parents’ Perceptions on the Debated Parenting Practice of Cognitive Enhancement in Healthy Children and Adolescents

**DOI:** 10.1007/s41465-022-00243-w

**Published:** 2022-06-15

**Authors:** Kati Hiltrop, Sebastian Sattler

**Affiliations:** 1 Institute for Sociology and Social Psychology, University of Cologne, Cologne, Germany; 2 Center for Health Communication and Health Services Research (CHSR), Department for Psychosomatic Medicine and Psychotherapy, University Hospital Bonn, Venusberg-Campus 1, 53127 Bonn, Germany; 3 Faculty of Sociology, University of Bielefeld, Universitätsstrasse 25, 33615 Bielefeld, Germany; 4 Pragmatic Health Ethics Research Unit, Institut de Recherches Cliniques de Montréal, Montréal, Canada

**Keywords:** Cognitive enhancement, Stimulant misuse, Moral attitudes, Parenting, Qualitative research, Content analysis

## Abstract

First evidence shows that some parents engage in the health-endangering practice of (mis-)using prescription drugs to boost their children’s school performance. But little is known about parental perspectives on this phenomenon. This study aims to better understand parents’ perspectives on the non-medical use of prescription drugs to improve healthy children’s cognitive functioning. We conducted twelve semi-structured face-to-face interviews with a diverse sample of parents in Germany, and applied qualitative content analysis to explore their perspectives on instrumentalizing prescription drugs for improving the performance of healthy children, including their underlying knowledge (gaps), moral evaluations, evaluations of accompanied risks and benefits, opinions on potential motivators, and wishes regarding policy-making. The results show that parents typically believed themselves knowledgeable about such prescription drug (mis-)use, although they were not aware of anyone in their social environment taking them for enhancement. Parents generally considered such behavior to be morally reprehensible, cheating, and similar to doping in sports, and they typically claimed that no situation or occasion could motivate them to administer prescription drugs to their healthy children. Health risks (including side effects or addiction) were a typical expectation of drug use. That doctors should give such drugs to healthy young people was seen as unjustifiable. The results suggest that morality and risk–benefit evaluations of parents play a major role in their decision-making concerning this potentially risky instrumentalization of non-medical drugs. These insights are of distinct importance, especially for future research and further discussions on this topic, such as an evidence-based public dialog and ethics debates.

**Supplementary Information:**

The online version contains supplementary material available at 10.1007/s41465-022-00243-w.

## Introduction

Parents have high expectations of their children’s performance in school and extra-curricular activities (Doepke et al., 2019; Nadesan, 2002; Wall, 2010). They are motivated by the desire to prepare for their children’s futures and often have a competitive mindset to exceed developmental norms. To meet these expectations, parents increasingly engage in intense parenting practices such as private tutoring (Wells et al., 2016). Researchers also highlight the susceptibility of parents to rationalize the non-medical use of prescription stimulants as an instrument to achieve their parenting goals, a relatively new debated and potentially health-endangering practice (Arria & DuPont, 2010; Sattler et al., 2021). Such non-medical use of prescription drugs specifically aimed at enhancing “mental functioning beyond what is necessary to sustain or restore good health” has been termed *cognitive enhancement* (CE) (Dresler et al., 2013, p. 29). While CE includes a wide array of further strategies, such as non-substance–based forms of enhancement (e.g., brain stimulation or brain computer interfaces), our study focuses on pharmaceutical CE since it has received increased attention in research and the media over the last years (Flanigan, 2013; Graf et al., 2013; Partridge et al., 2011; Racine et al., 2021; Sattler, 2020; Schäfer, 2018; Schleim & Quednow, 2017). Because of this, parents most likely know prominent substances used in this context, such as methylphenidate (e.g., Ritalin), amphetamines (e.g., Adderall), and modafinil (e.g., Provigil), which are usually prescribed for attention-deficit/hyperactivity disorder (ADHD) and sleep disorders.

First studies show that parents accept the risks of prescription stimulants in exchange for good grades not only for children diagnosed with ADHD (Fiks et al., 2013), but also for children without a medical condition (Cutler, 2014; Forlini & Racine, 2009). Parents are responsible for young people’s health behavior, including prescription drug misuse, since the latter depends on their parents’ stewardship and the resources they control (cf., Arria & DuPont, 2010; Coleman, 1994). Parents also serve as role models for their children’s socialization. Thus, an intergenerational transmission of the evaluation of the risks, benefits, and morality of prescription drug use is possible.

While the body of pharmaceutical CE research on other populations (e.g., university students or employees) is growing (see review in Schelle et al., 2014), the extent of parental support and concern regarding this potentially health-endangering practice as well as the underlying drivers leading to CE use have hardly been researched. This phenomenon and the accompanying problems may become more prevalent (Forlini & Racine, 2009; Singh & Kelleher, 2010) as scholars predict that CE-drugs will find their market “because parents want the best for their child” (O’Connor & Nagel, 2017, p. 5). Although it is unknown how frequently parents accept or foster CE-drug use in children, researchers warn that the misuse and abuse of prescription drugs “is one of the fastest growing drug epidemics in the United States, particularly among adolescents” (Conn & Marks, 2014, p. 257). In the USA, more than 1 million (4.8%) adolescents aged 12 to 17 misused psychotherapeutics in 2018, of whom 369,000 misused prescription stimulants (Substance Abuse & Mental Health Services Administration, 2019). Frequent motives for use include the desire to improve concentration and alertness, both of which can be considered CE (Teter et al., 2005).

### Concerns about Parents’ Engagement in CE-drug Administration and the Need for Further Studies

Sociologists, pediatricians, and ethicists are concerned that engaging in such practices may cause contagion effects (i.e., turning non-users into users), diminish freedom of choice, undermine authenticity and thus the child’s personality, violate children’s rights (plus propel competition in school), and undermine fair performance assessments between users and non-users (e.g., Gaucher et al., 2013; Sattler & Singh, 2016). Young people could be especially vulnerable to direct and indirect coercion by parents and peers. Since young people’s decision-making capacity is still developing, they may have difficulty assessing the risks of CE-drug use (Gaucher et al., 2013; Singh & Kelleher, 2010). Long-term health consequences include potential harmful effects on their developing bodies and brains, while acute exhaustion, abnormal heartbeat, or addiction can be more immediate consequences (Bray et al., 2004; Winder-Rhodes et al., 2010). Moreover, compared to legal forms of enhancement, such as energy drinks, accessing prescription drugs is often associated with morally questionable or illegal behavior, such as feigning symptoms or stealing the medications from friends, family, and other sources (Novak et al., 2007; van Veen et al., 2022). Given these negative consequences and due to the existence of more acceptable non-substance based options, pharmacological CE has therefore been considered legally and ethically unequal to other methods of increasing cognitive performance, such as private tutoring or coffee (Gaucher et al., 2013; Racine et al., 2021; Sattler, 2020). Based on this assessment, it can be argued that pharmaceutical CE — as one form of excessive parenting — has particularly negative consequences compared to other forms that are already often associated with physical and emotional harm for children (Miano and Palumbo, 2021; Oros et al., 2017; Pistella et al., 2021; Segrin et al., 2015). Further social and health concerns are summarized in a position paper endorsed by the American Academy of Neurology, Child Neurology Society, and the American Neurological Association (Graf et al., 2013). This paper is also critical about problems related to physicians’ professional integrity, e.g., their obligation to refuse inquiries involving improper drug use. Given these issues, most scholars agree on preventing, regulating, and monitoring CE-drug use in young people, especially children, and even suggesting legal sanctions for untrained parents administering CE-drugs without supervision (Gaucher et al., 2013; Graf et al., 2013). However, proponents of CE claim that CE-drugs qualify as tools to compensate for social disadvantages resulting from understaffed or overcrowded schools (Flanigan, 2013; Ray, 2016).

Investigating whether the realities and normative perceptions among non-expert stakeholders mirror the claims of experts could enrich the quality of this debate and the normative analysis (Forlini & Racine, 2012; Lucke, 2012; Sattler & Wörn, 2019). Including the interests of different stakeholder groups in society, like parents, is essential for democratic decision-making and to prevent potential negative consequences. Otherwise, the diverging views in the debate and often optimistic accounts in the media might create a challenging influence for the non-expert decision-making about whether to engage in or accept such behavior (Forlini & Racine, 2010). Therefore, we need a better understanding of parents’ knowledge, motivations, and thoughts on perceived consequences, as well as their moral considerations and justifications regarding CE (Forlini & Racine, 2012; Sattler & Wörn, 2019). Exploring such crucial factors of (health) decision-making (Carpenter, 2010; Cutler, 2014; Judson & Langdon, 2009) is also pivotal for empirically informed development and adjustment of health policies and intervention.

### Existing Research on the Parents’ Perspective

First qualitative research in Canada shows that parents are afraid using drugs for CE could become the “standard”, driven by a focus on achievement and noxious levels of competition in school, which they fear could undermine autonomous decisions not to use such drugs (Ball & Wolbring, 2014; Forlini & Racine, 2009). Ball and Wolbring (2014), however, found that some parents would approve of CE to avoid disadvantages for their children and to improve their grades and overall well-being, if the drugs were safe and efficient. Currently, most parents perceive CE as dangerous for their children and feel responsible to avoid harm (Ball & Wolbring, 2014; Forlini & Racine, 2009). Nevertheless, a recent study involving U.S. American parents found that information on social media about the benefits of CE-drugs can increase parents’ willingness to give such drugs to children (Sattler et al., 2021). Further, if others engage in such practices, the perceived pressure to act similarly can increase (Maher, 2008). Several parents said CE infringed on fairness and legality, and some felt such social pressure could overturn their values (Forlini & Racine, 2009). However, parents also voiced their disrespect for those who pressured their children into taking CE-drugs (Ball & Wolbring, 2014). In line with this, a survey found that over three-quarters of 710 parents surveyed supported policies preventing the abuse of CE-drugs in middle and high schools and believed schools should discuss its dangers (Davis et al., 2013; cf., Maher, 2008). Conversely, parents in another study believed CE should be a free choice and socially accepted, but users should be responsible for the consequences of their actions (Forlini & Racine, 2009).

Though parents are important stakeholders with legal responsibility regarding CE among their children and adolescents (Graf et al., 2013; Sattler & Wörn, 2019; Singh & Kelleher, 2010), research on their perspective is scarce and the very few pioneering studies reveal partially inconsistent findings. Since these studies mainly date back to the beginning of the last decade, it is an open question whether the stability of the results can be assumed over time for this relatively new behavior. This has led scholars to call repeatedly for further exploration of the CE phenomenon from different perspectives to understand the motivations for, goals of, and contexts in which CE may arise in young people (Ball & Wolbring, 2014; Sattler & Wörn, 2019; Singh & Kelleher, 2010).

The little existing research on parents’ perspectives is also limited to North America, and these findings may not be transferable to other regions due to cultural specificity (Bell et al., 2013; Lucke, 2012). One indication of this specificity can be seen in the variability of the prevalence of CE-drug use across 15 countries (twelve in Europe plus the USA, Canada, and New Zealand) for individuals aged 16 to 65 (Maier et al., 2018). For example, the 12-month prevalence rate of CE through prescription stimulants was 21.6% in the USA and 12.5% in Canada, while, in Europe, only The Netherlands and Belgium reached rates above 10%; rates were much lower in the three German-speaking countries (Germany, 3.0%; Switzerland, 2.6%; and Austria, 2.3%). Such country differences might reflect country-specific drug policies, diagnostic frameworks (e.g., regarding ADHD), prescribing behavior by physicians, and also different attitudes toward drug use (Lucke, 2012; Maier et al., 2018). Adderall, for instance, is an approved drug for ADHD in the USA, but in Germany, it is illegal (Sattler, 2016).

Germany is an interesting case because the prevalence of ADHD diagnosis, the total amount of prescribed ADHD drugs, and the mean amount of daily doses per case have substantially increased in recent years in young people, although lately are reaching a plateau (Langner et al., 2019). A share of this increase may have been influenced by CE practices, for example because of an observed over-diagnosing (Bruchmüller et al., 2012). An additional factor for this increase can possibly be attributed to parents’ involvement in such practices, but overall, knowledge about parents’ engagement in CE in Germany and their related risk-consciousness is lacking. Parents have not yet been investigated.

### Aim of the Study

This exploratory, Germany-based study aims to broadly investigate parents’ views on the use of CE in children and adolescents in Germany. We chose an explorative, qualitative design to examine parents’ perspectives on their knowledge and lack thereof about CE. We assessed parents’ estimated current and future prevalence as well as other contextual factors (e.g., competition in school) that can affect CE-decisions. Parents were also asked about the perceived risks and benefits associated with CE use. This included moral judgments and neutralization strategies of CE use as these factors can be further barriers to and drivers of this behavior. By probing parents to contrast CE to other methods for increasing school performance (e.g., private tutoring, energy drinks), we wanted to get a better understanding about how they perceive such action alternatives. Furthermore, we asked parents about their wishes regarding policy-making for CE.

## Materials and Methods

### Methodological Approach

We conducted semi-structured interviews, an approach that can help uncover information on emerging topics (including their context) and can thus create an in-depth understanding of parents’ practices, experiences, emotions, understandings, rationales, and justifications regarding CE (Ball & Wolbring, 2014; Bell et al., 2013; Cutler, 2014). Such interviews stimulated participants’ narrations without constraining their responses and enabled the investigator to ask for impromptu clarifications, while still guaranteeing comparability across respondents.

### Ethics Approval

The study and the recruitment strategy received approval from the Ethics Board of the Medical Faculty of the University of Cologne (Reference number: 17–040).

### Recruitment of Participants

The sampling of our study included parents from the federal state North-Rhine Westphalia in Central-Western Germany. Parents needed at least one child at school and living in their household. Such parents would be most qualified to answer the interview questions properly, assuming their children were exposed to conditions (e.g., performance assessment enabling social comparisons and competition) in which CE could become relevant. No distinction was made between biological and non-biological parents (e.g., birth parents versus adoptive- or step-parents).

We recruited a purposive convenience sample via personal and professional contacts of the first author (K.H.) (resulting in 7 participants) and snowball sampling (i.e., five contacts suggested by other participants). Potential participants were first contacted via phone to inform them about the study. Prior to the interviews, a screening survey assessed information on their socio-demographics (e.g., sex, age, marital status, number of children) and ratings of the importance of their children’s school achievements plus self-reported awareness of CE. Based on this information and the criterion of relevance (i.e., cases with the greatest potential to generate answers to the research question) (Flick, 2014), we selected a heterogenous sample (e.g., regarding parents’ gender, employment status, and age of children) of eligible participants for face-to-face interviews. The sampling process was stopped after twelve interviews, when parents’ narratives continued to repeat, which is known as data saturation (Grady, 1998, p. 26; cf., Saunders et al., 2018). Table 1 describes the sample characteristics.

**Table 1:** Sample characteristics (Number of observations = 12)

	n
Gender	
• *Female*	8
• *Male*	4
Marital status	
• *Married (living with spouse)*	10
• *Married (living separated from spouse)*	1
• *Single*	1
Education^1^	
• *Low*	2
• *Medium*	6
• *High*	4
Employment status	
• *Full-time*	5
• *Part-time*	4
• *Not working*	3
Self-reported awareness of CE^3^	
• *No*	6
• *Y*es	6
	Mean ± SD
Age (in years)	44.50 ± 4.36
Number of children	2.00 ± 0.43
Age of children (in years)	11.50 ± 3.83
Importance of children’s school performance^2^	6.75 ± 2.14

*Notes:* SD = Standard deviation; ^1^ Levels built according to the International Standard Classification of Education (ISCED): low (0–2), medium (3–4), high (> 4); ^2^ Assessed with the item “How important to you is good school performance in your child(-ren)?” measured on a scale from 1—very unimportant to 9—very important; ^3^ Assessed with the item “Have you heard of medications being given to children and adolescents to enhance their cognitive performance — even though there is no medical need? Response options: “No” and “Yes”

After informing participants about audio-recording, pseudonymization, data usage, and voluntariness, informed consent was obtained. The interviews lasted between 20 and 40 min. Participants could choose between cash incentives or donations to UNICEF (both 15 Euros, approximately $17.25 USD).

### Informational Material

Parents received informational material to ensure basic understanding of the topic and encourage conversation, even when they had lower levels of knowledge on pharmaceutical CE (see also Bell et al., 2013; Forlini & Racine, 2009; Partridge et al., 2013). Our material (available to the reader upon request) included a definition of CE, and potential CE methods such as activities like meditation and sports, substances that are freely obtainable or available only in pharmacies (e.g., ginkgo biloba, caffeine pills), and prescription drugs (e.g., methylphenidate). All information given was based on scientific studies and double-checked to avoid influential language or biases due to selection. This information and the interview guide (see next section) were reviewed by a CE expert and tested in two pilot interviews, which helped add relevant questions and adjust formulations.

### Interview Guide

The semi-structured interviews covered a broad set of six topics regarding CE identified during the literature review: knowledge about CE; its estimated prevalence; motivations to use CE; moral evaluations and justifications of CE; comparisons of CE with other methods; consequences of using it; and estimated future developments. The interview guide contained open-ended questions for these topics, including optional questions for less talkative participants (Table S1, Online Supplements).

### Data Analysis

The audio-recorded interviews were transcribed verbatim. Content analysis was used to find recurring themes in the collected material (Mayring, 2015), and were derived inductively from the interviews. The coding in segments of sentences and paragraphs was carried out by the first author (K.H.). While coding the first interview, a code plan was developed, refined and supplemented by analyzing subsequent interviews. Both authors then structured the (sub-)domains and themes by assigning similar themes to categories. The (sub-)domains were derived deductively from existing literature and make up the topics of the interview guide. The data were again coded by two additional people to ensure analysis reliability. Differences in coding were discussed until consent was reached and approved by the second author (S.S.). Finally, the themes were grouped into three categories — “general” (11–12 participants), “typical” (6–10), and “variant” (3–5) — based on the number of participants who mentioned them, while themes expressed by fewer than three participants were not reported (Bell et al., 2013; Hill et al., 2005; Partridge et al., 2013). Professionally translated quotes from the parents were used to illustrate our findings. Duplications and filler words were omitted to ease understanding.

## Results

The content analysis resulted in a broad set of 50 themes (Table 2). Based on their frequency of occurrence, six were labeled as “general”, 28 as “typical”, and 16 as “variant”. Depending on their content, these themes were clustered and summarized into six thematic domains (e.g., “evaluation of drug effects”) and 16 sub-domains (e.g., “evaluation of efficacy for CE”), while the themes mainly mirrored topics of our interview guide (Table S1), along which we will present the results.

**Table 2:** General, typical, and variant (sub-)domains with themes and frequencies based on the qualitative content analysis of parents’ views on CE

Domains, sub-domains, and themes	Frequency (absolute)
**Parents’ knowledge about CE**	
*** Existing knowledge***	
Parent has knowledge about CE-drugs	Typical (9)
Aware of nutritional enhancers	Variant (4)
*** No knowledge***	
Parent has no knowledge about CE-drugs	Variant (3)
**Estimated current and expected future prevalence**	
*** Drug use for CE***	
Parent does not know persons using drugs for CE	General (11)
Parent expects an increase of CE	Typical (10)
Parent refuses future willingness to use CE	Typical (10)
Prevalence estimation of CE ≤ 5%	Typical (8)
*** CE-drugs used for treatment***	
Parent knows persons using CE-drugs for treatment of disease	Typical (8)
Criticizing the (over-)diagnosis of ADHD	Typical (6)
Parent does not know persons using CE-drugs for treatment of disease	Variant (3)
**Motivating factors for and against CE-drug use**	
*** Personal motivation***	
No situation or occasion motivates use	Typical (10)
*** Motivation of others***	
Performance pressure and strict requirements in school	Typical (10)
Laziness	Typical (9)
Competition	Typical (6)
Unfavorable conditions in the school system	Typical (6)
**Evaluation of drug effects**	
*** CE-drug efficacy***	
Expectation of more success in school	Typical (8)
Expectation of increased concentration	Typical (6)
Doubts about better performance in healthy young people after using CE-drugs	Typical (6)
Expectation of increased receptivity	Typical (6)
Expectation of negative effect on learning motivation	Variant (5)
Expectation of decreased nervousness	Variant (4)
Beneficial effects are limited to time of intake	Variant (4)
Expectation of a different effect on healthy and sick people	Variant (4)
Expectation of positive effect on learning motivation	Variant (3)
*** Potential side effects of CE***	
Expectation of addiction/dependency	Typical (10)
Expectation of physical side effects	Typical (8)
Expectation of psychological side effects	Typical (8)
Uncertain expectations about side effects	Typical (6)
*** Other effects of CE***	
Stigmatization/decreasing popularity of children among peers in school	Variant (5)
Young people may support CE in case of success	Variant (3)
**Moral assessment**	
*** Disapproving views***	
CE is morally reprehensible	General (11)
CE is cheating/unfair	General (11)
Getting prescriptions for CE-drugs from doctors does not justify their use	General (11)
Approval of prescription drugs for ill people does not justify use among healthy young people	Typical (10)
Prescription drug use is only morally acceptable for treatment of young people with an illness	Typical (9)
CE is not morally acceptable for own child/ren in any situation	Typical (9)
Performance of pupils is not comparable if some use CE	Typical (9)
Riskier enhancement methods (e.g., illegal drugs) do not justify CE-drug use for young people	Typical (7)
Parent criticized other parents willing to use CE for their child/ren	Variant (5)
*** Neutral views***	
CE is not cheating/unfair	Variant (4)
Performance is comparable when some pupils use CE	Variant (3)
*** Need for intervention***	
Parent saw need for action from professionals due to moral concerns	Typical (8)
**Comparison of prescription drugs with other methods**	
*** Comparison to illegal drugs***	
Parent evaluated comparison of doping and CE as adequate	General (11)
CE among healthy young people is objectionable as illegal drug use	Variant (4)
*** Comparison to tutoring***	
Private tutoring and CE-drugs are not comparable	Typical (10)
Private tutoring only leads to success achieved through work	Typical (9)
*** Comparison to energy drinks***	
Energy drinks and CE-drugs are not suitable for children	Typical (7)
Energy drinks have a weaker effect	Variant (4)
Energy drinks are different due to being freely obtainable	Variant (4)
*** Preference for non-medical strategies***	
Parent preferred non-medical alternatives over CE	General (11)

*Note: N* = 12 participants total; general themes were named by 11–12 participants, typical themes by 6–10 participants, variant themes by 3–5 participants

### Parents’ Knowledge about CE

#### Existing Knowledge

A typical theme was that parents attributed themselves to be knowledgeable about pharmaceuticals for CE purposes. This knowledge mainly stemmed from the usage of CE-drugs for therapeutic reasons. Few parents reported learning about such drugs through their studies or job. One parent of two children (P1), working in the healthcare sector, explained:

“I know from my job that many high school students and recent graduates would like to take certain medications or have them prescribed by a doctor, like for example Ritalin or Medikinet, these are the most common ones I’ve heard of.”

In addition to pharmaceuticals, many parents were aware that certain nutritional aids (such as dextrose, caffeine-based drinks, or energy drinks) may enhance cognitive performance. This was underlined by a statement of a mother (P5): “Yeah, so normal stuff before tests or during tests with dextrose and stuff like that, that’s the usual. We used to do that to boost the concentration a bit.” Thus, knowledge also exists through personal experience from consuming such marketed products.

#### No Knowledge

A minority of parents was not at all familiar with the fact that cognitive performance can be targeted with medication in healthy individuals.

### Estimated Current and Expected Future Prevalence

#### Drugs used for CE

The parents considered CE to be uncommon (typically at 5% usage rate or lower) among young people in Germany. They most likely extrapolated this information through their own (in-)experience and knowledge regarding the use of such drugs for treatment purposes. While some parents expected the prevalence to increase in the future, possibly even reaching “an untenable state” (P2, a father who knew about CE before the interview), almost all parents refused to be part of such a trend.

#### CE-drugs Used for Treatment

Some of the parents viewed the rising incidences of over-diagnosis of ADHD to be a driver of this trend and critiqued the medicalization of CE. A married stay-at-home mother of two (P4), suspected, “that some parents maybe don’t take care of their children enough and that it’s easier for them to drag them to the doctor and stick them in a pigeon-hole.” For this mother, going to the doctor was viewed as an easy route for parents to take when they did not want to put in much effort in their child’s life.

### Motivating Factors for and Against CE-drug Use

#### Personal Motivation

Parents typically could not imagine any potential situation or condition that would motivate them to give CE-drugs to their children. A mother for whom school performance was relatively important (P1), described:

“I absolutely can’t say if there might be a situation where I would give my child [CE] medication. Why? I can’t think of—even if it was only Smarties, which taste good and have no side effect—I just don’t know, no.”

This mother’s view on not seeing any rationale for engaging in CE and a strict refusal of CE medication was prevalent in our sample. She and other parents would not even consider CE in the absence of side effects. When probing specific situations that might encourage administering CE-drugs to their children — such as performance requirements, laziness, academic competition, or unfavorable conditions like large classes within the school system — most parents denied such conditions existed.

#### Motivation of Others

Some parents assumed that other parents might be affected by the above-mentioned circumstances: “Yeah, I would assume that it [the pressure to perform in society] exists, yeah. But for me personally, it would be out of the question, no. But I suppose there are parents who would use these substances” (P2). Even when refusing CE drugs for their own children, this example illustrates that performance pressure could motivate other parents to act differently.

### Evaluation of Drug Effects

When asked about possible outcomes of CE-drugs, parents were concerned about *CE-drug efficacy*, *potential side effects*, and *other effects of CE-drug use*.

#### CE-drug Efficacy

Views on the efficacy of CE-drugs diverged. The most frequently expected effect of CE-drugs was a short-term improvement in school performance, and also enhanced long-term prospects in the job market. Parents typically believed cognitive functions like concentration and receptivity can be improved by using CE-drugs. Few parents expected CE drugs to reduce nervousness in the classroom, but meanwhile the improved attention and consequently better performance would motivate children to continue learning: “If the child notices ‘Hey, I can pay attention better, follow better [in school]. I can maybe do my homework better and then automatically get better grades’. That it [child] is maybe more motivated to go to school” (P1). Thus, this parent expected behavioral changes in addition to immediate effects on cognitive functions.

Opposing views about CE-drug efficacy were voiced by half the parents. A father of three who knew about CE before the interview (P12), explained other conditions were more relevant to a child’s performance than medication: “I think there are so many different circumstances that I can’t really imagine the drugs or medications have such a huge effect.” In this quote, he referred to nature and nurture (e.g., intelligence, character traits, family, and school) having much stronger effects on school performance than drugs.

A typical worry expressed was that CE-drugs might have a negative impact on learning motivation. The CE-drugs were expected to have an effect at the psychological level, in which students [or users] would not feel the need to put forth much effort into tasks because the medications were doing the work for them: “Yeah, maybe that they [the children] say ‘okay, I’ll do it more-or-less through the medications [*laughs*]. I don’t really need to make any real effort anymore’” (P12). Consequently, the interviewee expects that CE undermines motivation in the long term. Another concern was that drug effects are limited to the duration of consumption: “If you discontinued them [the medications], they [the children] would not be as concentrated anymore, nervous, easily distracted and so on, and with the medication they’re simply in a better state of mind [*laughs*]” (P3). A further worry regarding drug efficacy was that CE-drugs might work well in the therapeutic contexts for which they were designed, but might have different effects on healthy people.

#### Potential Side Effects of CE

Parents mentioned various side effects, including physical and psychological consequences. Although one can distinguish between psychological and physiological addiction and dependence, a clear distinction could not be made in the parents’ statements. Due to the frequent emergence of the theme of addiction and dependency (10 out of 12 parents), this was coded separately from other side effects.

A single father of one (P10), connected dependency to an inability to invest in long-term effort:

“It also becomes like dependence, actually. That in future situations, too, you’ve learned ‘I can fall back on that’ [CE medication] and then maybe I do it more often in the future and don’t fight my own way through a situation […]. And this ability to dig one’s own way through—maybe the children can’t even acquire it then.”

He describes his fear that children would come to see CE drugs as a safety net. They would not fear failure or be motivated to put in true effort in their studies because the drugs could be a route to “easy success”. This could prevent the children from learning resiliency in overcoming challenges not only in academics, but also in valuable life skills.

Other anticipated psychological side effects of CE were, inter alia, changes in personality or self-esteem, including a suppression of *true* self: “The children are so calm and sedate only because they’re taking this medication, but the actual personality doesn’t even emerge because they’re always buffered below this level of substance, like in cotton padding, I think” (P8).

Typically expected physical side effects included damage to the brain, organs, or vessels, and fatigue. Some parents were skeptical about physical long-term health consequences: “I don’t know right now what the effects are on the body, on the brain, long-term, if any damage occurs, that they [CE drugs] maybe damage some kind of cells or something” (P1). While raising concerns about potential long-term effects, a mother, who was aware of CE before the interview (P4), mentioned that knowledge about CE-drugs was poor due to the lack of long-term studies, which increased the danger of using them:

“I can definitely imagine that some kind of physical damage could happen at some point [if the drugs are taken for longer]. […] But there are never long-term studies that could then say that ‘such and such an effect will then occur later,’ and this is the danger.”

Half of the parents admitted uncertainty about potential side effects due to their limited medical knowledge.

#### Other Effects of CE

Stigmatization as a consequence of consuming CE-drugs was a variant theme. Parents expected that teasing or social exclusion might occur if children discovered the use of CE-drugs within their social groups: “It [CE-drugs] can become an instrument of power among the kids, where they constantly tease each other or play around, and are forced to make social distinctions and get excluded” (P10). This father perceived CE-drug use to become a signal upon which children are discriminated against and are excluded from friendships.

Parents also worried about positive drug experiences (i.e., improved learning), which might lead young people to develop positive attitudes toward CE-drugs and they might use them more: “When children realize that of course they can study better and more easily with the help of a medication, I can totally imagine that they see that as good.” (P4). This mother thinks that such positive attitudes might promote the devaluing of other strategies to solve problems.

### Moral Assessment

There was a general agreement regarding the moral assessment of pharmaceutical CE, as indicated by three general and six typical themes.

#### Disapproving Views

The majority of parents considered CE in young people as “morally reprehensible” and “not okay” (P1). When asked about situations or occasions (real or imagined) that might justify administering CE-drugs to their healthy children, parents typically refused, as a mother who valued the school performance of her two children (P7) said, “So, I don’t know if in this regard my thinking is simply too radical. For me it’s so farfetched, the idea that my children would take these kinds of medications. I can’t name a single example.” This mother was apologetic for her strong refusal and was worried about the morality of pushing her children to these new limits.

Parents generally described CE as unfair because it can be viewed as cheating. Two dimensions were mentioned: unfairness because of relative disadvantages for classmates and unfairness because young people were exposed to side effects:

“Yeah, it would be unfair to the other children [who don’t use CE]. But I think it’s most unfair to the children who take it. I actually find this much more regrettable and much more unfair, because these children simply are harmed.” (P5)

A typical explanation for parents’ perceptions of unfairness referred to the challenge (e.g., for teachers) in comparing performance between users and non-users: “In some circumstances you don’t know who’s taking medication and how can you compare who’s taking which medication, in which dose and what influence this has on their normal performance” (P2). This statement reflected a belief in the existence of and difficulties in assessing *true* performance (an analogy to the *true* self).

Parents also showed disapproval toward parents willing to administer CE-drugs. As P7 put it: “No one could be that stupid.” Most parents considered prescription drug use legitimate only for treatment purposes, but even here they were cautious: “Maybe it’s okay with ADHD, you’d have to see how the children are and maybe I’d try it, depending on what the specific kid’s like. But for healthy kids, no, I wouldn’t do it. No way” (P3). Such disapproval remained when different scenarios or justifications for CE use were introduced, such as situations where medication was approved for certain diseases and riskier methods (like illegal drugs) were used for CE. Often parents argued a fear of side effects: “If a healthy person takes a medication that is actually only available on prescription, there may be side effects. I don’t think someone who’s healthy should take that risk” (P7). This parent took the legal status of the drugs as an indicator of the seriousness of side effects and for being cautious.

Despite the legality of physicians prescribing drugs to healthy young people, this did not alter the negative attitudes toward CE in some of the parents. A married mother of two who was unaware of CE before the interview (P9), questioned the morals of such physicians and called for legal action: “…actually that doctor should lose their license. […] I think they should be taken to court.” This statement underlines parents’ strong moral objection against a behavior seen as dangerous.

#### Neutral Views

Views not clearly disapproving of CE were in the minority. Some parents stated that they did not perceive CE-drug use as unfair or cheating. One parent said that they would not feel cheated if other children took CE-drugs, but suspected that parents who administer CE engage in some form of self-deception:

“I would hope that my kid’s grades would stay the same in any case [of other students using CE drugs] and maybe only the other kids’ grades would get better and so it wouldn’t matter to me. This is why my first thought is that it’s not basically unfair. […] I actually don’t see this as cheating us. If anything the people might be cheating themselves.” (P12)

From his point of view, CE is not an unfair practice since the achievements of his children are not diminished if others improve their grades through CE, referencing absolute grading (but neglecting grading on a curve). Thus, this father perceives achievements in absolute terms and thinks that a student’s competition is internal. Therefore, students who use CE are cheating themselves by taking the “easy way out” to get good grades.

Moreover, few parents, like a father of three (P12), even expected students’ performance to remain comparable by describing CE-drugs as one of many contextual factors affecting it:

“Say that one kid comes from a home with intellectual parents and one kid doesn’t come from a home with intellectual parents, how should I compare their performance? I do this in fact on the basis of their grades. It’s simple […] For me, taking medication would be another determining factor that changes the kid’s performance.”

Similar to highly educated parents passing their knowledge and human capital to their children, CE-drugs are seen as no different in adding to the complexity of performance assessments.

#### Need for Intervention

Another common theme was the call for intervention due to concerns around the current political and educational systems in place. Some parents called for immediate action to impede the expansion of CE; others saw less urgency:

“If it [CE drug use] becomes commonplace, […] if it becomes a social question, then it also becomes politically relevant. Because […] maybe something is wrong with the system, maybe the standards are too high, the goals are too ambitious or the [school] requirements.” (P9)

According to the parents, the first professional group to intervene should be teachers and doctors. The school system and politicians were also seen as accountable to reconfigure performance expectations and reduce the perceived need to use CE-drugs. However, within these statements, it became clear that political action should only take place when CE has become a general phenomenon.

### Comparison of Prescription Drugs to other Methods

Parents were explicitly asked to compare CE prescription drugs to tutoring, energy drinks, and illegal drugs.

#### Comparison to Illegal Drugs

A general pattern was that parents described CE-drugs as being most similar to illegal drugs. They mainly argued that the intake of both types of substances enabled achievements that would be impossible without them, as P5, a mother of two, described it: “So, in both cases something is taken to achieve something that you normally wouldn’t. I do think they’re very comparable, yes.” By making this comparison, CE-drug use by young people was considered similar to doping in sports: “I would compare it to [doping] among athletes, doped kids, doped athletes” (P2). Thereby, this parent equates the practices of substance misuse with the illegality, unfairness, and dangers of sports doping that is implied in “doping” kids.

#### Comparison to Energy Drinks

Although similarities between CE-drugs and energy drinks were acknowledged less frequently, parents typically described both methods as unsuitable for children. Neither belonged in children’s bodies and were harmful: “I absolutely disapprove that because they [energy drinks] can also cause significant side effects and can even lead to bodily harm and so we don’t allow our children to consume them.” (P4) Some parents also acknowledged differences between CE-drugs and energy drinks, since energy drinks were assumed to have a weaker and shorter effect than pharmaceuticals. Additionally, parents referred to the legal status of both types of substance:

“I also tried one of these energy drinks and it didn’t kill me. It’s similar with the medication but for me there’s a difference of degree. Something being freely available also suggests to me that it’s not quite as harmful as things that aren’t freely available.” (P11)

According to this parent, the widespread availability of energy drinks was interpreted as an indicator of less harm in comparison to prescription drugs.

#### Comparison to Tutoring

Tutoring and CE-drugs were typically evaluated as distinct methods due to the different motivations behind them and the inherent features of these methods, as for this mother (P5):

“I think that’s what tutoring is for, to compensate for deficits in the subject matter, things that may have been neglected, that weren’t understood, that were missed, to simply go over these things again and make them comprehensible to the kids. In contrast, I think these medications target only concentration. And concentration is not produced by tutoring, for that I think other things are more important, like balanced afternoons, sports, simply a counterbalance to school.”

She highlights that private tutoring requires effort to improve a deeper understanding, while it cannot help to increase concentration as CE-drugs directly do. Parents also classified private tutoring as “the normal way to improve performance” (P2).

#### Preference for Non-medical Strategies

Generally, parents preferred “normal” and non-medical methods (i.e., private lessons, repeating a school year, or using dextrose or coffee) over energy drinks or drugs.

## Discussion

### Summary and Reflection on the Results

While the investigation of parents’ perspectives instrumentalizing prescription drugs for improving the performance of healthy children is limited (especially in Germany) and has only revealed partially consistent findings, we explored a broad variety of facets regarding this potentially health-endangering parenting practice. This exploration included parents’ underlying knowledge and its gaps, moral evaluations, evaluations of accompanied risks and benefits, opinions on potential motivators, and wishes regarding policy-making. Besides discovering novel aspects regarding parents’ views on CE, several of our findings support the few previous research endeavors with parents from North America and CE-research in other populations (such as students). This lends credit to our study and underlines the convergence of some perspectives on CE.

A finding that mirrors initial non-representative survey research of German pupils on the use of prescription drugs for CE purposes (Franke et al., 2011), is that the interviewed parents viewed CE among young people in Germany as rather uncommon (≤ 5%). Parents’ prevalence estimates might be influenced by their lack of personal experiences with CE and not knowing others who give CE-drugs to healthy young people. These types of estimates are highly subjective given that CE is a practice that is usually not publicized. Still, some parents had the subjective expectation of that the dissemination of CE drugs would increase, which was based on the belief that pharmaceutical CE would spread in the future and through the perception that ADHD is over-diagnosed. This expectation exists in professionals (Singh & Kelleher, 2010) and is in line with a study that suggests that over-diagnosing in Germany occurs (Bruchmüller et al., 2012). While such expectations may not match objective prevalence rates, subjective perceptions about a high or increasing prevalence can have behavioral consequences and if some parents start administering drugs to their children for academic purposes, it can create a contagion effect that reinforces the subjective prevalence beliefs (Huber et al., 2022).

However, parents in our study were typically critical about using CE-drugs for their own children. They could not think of any circumstances that would motivate them to use CE, including indirect coercion through other parents’ use. When probed about specific motivators or rationalizations relevant in the ethics debate (Flanigan, 2013; Ray, 2016) or other studies (Cutler, 2014; Forlini & Racine, 2009) (such as performance pressure, competition, laziness, or unfavorable schooling conditions), parents admitted that other parents might be affected, but denied that they would personally be influenced. Parents from studies in North America also described perceiving the aforementioned conditions, but some named scenarios in which they would accept administering CE-drugs (e.g., if responsible intake helped children through exams or desperate situations, or promoted overall wellbeing) and admitted existing social pressures might overturn their opposition to such drugs (Ball & Wolbring, 2014; Forlini & Racine, 2009). Attitudes toward CE may vary across cultural and social contexts, as suggested by prior research that shows that the prevalence of CE-drug use in the general population is lower in Germany as compared to the USA (e.g., Maier et al., 2018). However, our results could also suggest that parents in this study provided socially desirable answers about their motivations due to discomfort of reporting behavior that may endanger their children’s health (see the “Strengths, limitations and directions for future research” section).

Due to parents’ general negative sentiments toward CE for children, even hypothetical CE-drugs with zero or unlikely side effects would not motivate the parents in our study to give such drugs to their children, while students have been more open to such practices (Franke et al., 2012; Sattler et al., 2013b). Parents considered pharmaceutical CE in healthy young people to be risky and feared potential harmful (long-term) physical and psychological side effects (Ball & Wolbring, 2014; Forlini & Racine, 2010, 2012; Graf et al., 2013; Singh & Kelleher, 2010). Therefore, a major theme was addiction and dependency. However, it is an important finding that half the parents indicated uncertainty about possible side effects of CE-drugs. This could be a reflection of their lack of experience with the drugs, as well as a gap in medical knowledge and health literacy.

Few participants voiced concerns about the little-investigated non-medical side effects (Ball & Wolbring, 2014; Sattler & Singh, 2016) such as the stigmatization and social exclusion of children who use CE-drugs by their peers. CE-drug-using children might be seen as being in need of the drugs and too weak to be able to compete, or as engaging in an unfair practice. Reference has been made to authenticity, since the true self and true performance might vanish if CE-drugs are used (Bell et al., 2013; Forlini & Racine, 2012). While several parents in our study envisioned positive drug effects (e.g., greater success, increased concentration and receptivity), they generally expressed more concern about negative non-medical effects of CE-drug use than possible beneficial effects.

Understanding the moral evaluation of CE behavior is important because morality is highly predictive for engaging in such behavior (Sattler et al., 2013b). Parents showed great consensus regarding the moral and ethical assessment of pharmaceutical CE. In general, the majority of parents evaluated CE as morally unworthy of praise, unfair, and cheating. This evaluation was apparently more persistent than in previous studies. Offering the parents different justifications (e.g., the drugs are safe or they are prescribed by doctors) given by stimulant-drug-using students (Cutler, 2014) did not alter their views.

A novel finding was that CE drug use was viewed as unfair to the children who are exposed to the side effects solely for the sake of fulfilling their parent’s wishes for them to excel in school, which adds another dimension to the argument that CE drugs are unfair due to the relative disadvantages they produce. In relation to these novel findings, achieving higher performance with CE was viewed as self-deception.

While most parents in our study viewed CE as cheating, parents in Canada seemed more neutral (Forlini & Racine, 2010). Nevertheless, some parents in our study found it difficult to evaluate CE as fair or unfair, possibly due to unfamiliarity with the concept or because they did not expect CE-drugs to affect others (thus causing no moral conflict) (cf., Pohl et al., 2018, who observed similar pattern in university students and employees). Interestingly, several parents would engage in informal social control and criticize other parents for engaging in such behavior. This informal social control has been shown to be widespread (Cancer et al., 2018) and reduce the willingness to use such drugs in university students (Sattler et al., 2014).

One means to deal with unfamiliarity with a certain practice such as pharmaceutical CE is “comparison to an allegedly better known phenomenon” (Forlini & Racine, 2010, p. 622). We asked parents to relate CE-drugs to illegal drugs, energy drinks, and private tutoring. The comparisons again reflected parents’ negative attitudes toward CE-drugs. The comparison with illegal drugs was seen as most appropriate (cf., Forlini & Racine, 2010), due to the unfairness associated with doping and how using CE drugs achieves similar goals. Comparisons with energy drinks were often disliked, and both were judged inappropriate for young people, although weaker and shorter-term effects were attributed to energy drinks. Parents viewed private tutoring as the most “normal” and “common” method to increase their children’s performance. They often indicated a preference for non-medical methods described as “normal” (i.e., private lessons, repeating a school year, or using dextrose or coffee) in opposition to energy drinks or prescription drugs. This mirrors ethics discussions (see Introduction) arguing that prescription drugs are legally and ethically unequal to other forms of enhancement, for example, due to their potential side effects, illegal obtainment, unfairness, undermining the importance of hard work, and a medicalization of societal problems (Gaucher et al., 2013; Racine et al., 2021; Sattler, 2020).

In the ethics debate, the question arose about when to take (political) action regarding CE (Sattler et al., 2013a). In our study, the majority of parents saw the need for intervention, but the urgency that teachers, schools, physicians, or politicians should take action varied. Still, if physicians would give CE-drugs to healthy young people, parents would call for legal reactions. This underlines the major point that most parents in our sample are very concerned about this practice.

### Strengths, Limitations and Directions for Future Research

As the first study on parents’ perspectives on CE in young people in Germany, this exploratory study aimed to provide a preliminary understanding of a broad set of themes regarding this topic in one federal state in Central-Western, Germany. The findings on parents’ evaluation of risks, benefits, and moral concerns associated with drug administration (factors that are relevant for health decision-making) (e.g., Carpenter, 2010; Cutler, 2014; Judson & Langdon, 2009; Sattler et al., 2013b), can inform further qualitative inquiry and large-scale research aimed at evaluating the representativeness of the opinions voiced. Comparative studies would be especially beneficial to examine if, how, and why parents in countries with higher prevalence rates of prescription stimulants (such as the USA, Canada, or the Netherlands) may systematically differ in their views toward young people using CE than parents in countries with relatively low prevalence rates (such as Germany, Austria or Portugal) (Maier et al., 2018).

Nevertheless, our study is not without limitations. One limitation is that our sample was a small, convenience sample that does not allow for generalized findings. Still, the sample size is comparable to similar studies and it provides first insights into parents’ perspectives (e.g., Aikins, 2011; Ball & Wolbring, 2014; Heyes & Boardley, 2019; Hildt et al., 2014; Vargo & Petróczi, 2016). While reaching out to heterogeneous parents during our purposive sampling, results showed that the parents’ views on CE in healthy children were rather homogenous. Further interviews did not create new insights and thus indicated data saturation (Flick, 2014; Grady, 1998; Saunders et al., 2018).

Due to the fact that our sample was recruited via personal/professional contacts and snowball sampling, parents may have tried to give socially desirable answers due to reputation concerns. It is also known that face-to-face interviews can result in socially desired responses (Krumpal, 2013). We, however, tried to create a non-judgmental and tolerant atmosphere to encourage parents to talk openly about their views contacts and snowball (cf., Vargo & Petróczi, 2016). The interviewees were able to choose a location they were familiar with (mainly in their home) and we ensured that the interviews were free from interruptions. Moreover, participants were explicitly informed about the pseudonymization of the data. Still, we encourage future research, especially quantitative studies, to employ anonymous assessment strategies to reduce possible social response biases.

Future qualitative and quantitative studies might look to explicitly compare parents’ attitudes toward different forms of substance-based enhancements, such as prescription drugs or nutritional supplements, with nonsubstance-based neurotechnologies, such as brain stimulation or brain-computer interfaces (Dresler et al., 2013; Schmied et al., 2021; Wagner et al., 2018). Such studies may also examine how these attitudes relate to respondent characteristics, such as political orientation, competitiveness, or social status.

Finally, it should be noted that all parents received informational material about CE prior to the interview to clarify the topic of the interview and to enable parents’ engagement in a dialog even if they have not heard about pharmaceutical CE before. This strategy of providing prompt material as stimulus is established in exploring public attitudes toward innovative topics (Bell et al., 2013; Forlini & Racine, 2009; Partridge et al., 2013). It cannot be ruled out, however, that this material influenced parents’ responses. We expect this influence to be minimal, because we aimed at very brief (less than a page) and factual basic information supported by research. The material was double-checked to avoid influential language or biases occurring due to selection of the information. Additionally, it was reviewed by an expert on CE and approved by the local ethics committee. Parents were encouraged to express their own opinions (Partridge et al., 2013). Future studies may, however, examine if parents without prior information may respond differently.

## Conclusion

This study has contributed to the limited research on CE among young people by providing valuable insights into the perspectives of parents. To our knowledge, this is the first study investigating parents’ views on CE in Germany. Knowing more about their perspectives is of distinct importance for informing public dialog, ethics debates, public health research, and policy-making. It demonstrates how these crucial stakeholders perceive and evaluate CE-drug use motivationally and morally, and it is useful for evaluating what knowledge (gaps) they have. These aspects are relevant because they affect parents’ decision-making and also reveal whether health research and ethical analysis miss crucial aspects or misunderstand parents’ perspectives. Our results reveal overlaps with the ethics debate, although parents’ knowledge on CE practices is limited; still, they have a relatively clear view on whether to engage in such practices in the future. While parents predicted an increase in the prevalence in the use of CE-drugs, parents showed a strong opposition to do so for their own children. This discrepancy can have methodological reasons (i.e., a social desirability bias as discussed above), but the marked refusal to do so might also be explained by a relatively concordant moral reprehension of CE-drug use in our sample, as well as fears of negative health consequences and ambiguity about the perceived effectiveness of such drugs. This highlights the importance of morality and risk–benefit evaluations in further theory-guided research, but also the need to examine larger samples aiming for representative and anonymous assessments of parents’ views on CE and the willingness to administer such drugs to their children. Further research should also examine the role of physicians as gatekeepers for CE and young people themselves when it comes to CE (Graf et al., 2013). It should consider these two stakeholder groups as well as parents to substantiate knowledge of CE in Germany and elsewhere to uphold child and family well-being and to protect against adverse childhood events (such as exposure to the side effects of medication). The need for such research and to monitor developments is underlined by predicted prevalence increases among scholars (O’Connor & Nagel, 2017; Singh & Kelleher, 2010) and non-experts in this study, as well as by hints that the prevalence in other social groups (e.g., employees) is already rising in many countries including the USA and Germany (Maier et al., 2018).

## Supplementary Information

Below is the link to the electronic supplementary material.

Supplementary file1 (DOCX 31 KB)

## Data Availability

The data that support the findings of this study are not openly available due to reasons of sensitivity. The research material has been extensively described in the manuscript and the Supplementary information.
