# Supplementary material for: Parents’ Perceptions on the Debated Parenting Practice of Cognitive Enhancement in Healthy Children and Adolescents
Source: J Cogn Enhanc. 2022 Jun 15;6(3):373–88. doi: 10.1007/s41465-022-00243-w (PMC9360129; doi:10.1007/s41465-022-00243-w)
Supplement: Supplementary file 1 — Supplementary file1 (DOCX 31 KB) [file 41465_2022_243_MOESM1_ESM.docx]

Supplementary Material

Table S1: *Topics of the interview guide and exemplary questions*

| Topic | Exemplary questions |
| --- | --- |
| Knowledge about CE | First of all I’m interested in what you already knew about cognitive enhancement among children and adolescents prior to this study and before you received the informational leaflet. |
| Prevalence of CE | How widespread do you estimate the intake of such drugs at schools is in Germany – in your perception, how many out of 100 healthy children take them? |
| Motivations to use CE | In the informational leaflet, we described drugs with the active ingredients methylphenidate and modafinil – maybe you remember them. What or which situations/occasions could induce you to use such drugs for the cognitive enhancement of your child/ren? |
| Moral evaluation of CE | Now we want to know about your moral evaluation of the enhancement of cognitive performance among children and adolescents with prescription drugs. “Morally” means something like ethical or in the broadest sense acceptable. |
| Justifications of CE | Under what circumstances or in which situations would you find it “okay” or morally acceptable that your child/ren should try to enhance their cognitive performance with prescription drugs? |
| Comparisons of CE with other methods | If children do not perform well in school, methods such as private tutoring are used to improve their achievements. How far, do you think, can the enhancement of cognitive performance with prescription drugs and private tutoring be compared or not compared? |
| Consequences of using CE | Now I would like to talk about the consequences the enhancement of cognitive performance might have. If you imagine giving such prescription drugs to your child/ren, what consequences would you expect in the short and long term? |
| Estimated future development of CE | Finally, a question about what could happen to the intake of enhancing drugs among healthy children within the next 5 to 10 years in your view. What development do you expect at your child’s school in the future? |

*Notes:* The interviews were conducted in German. The exemplary questions have been translated into English. Prior to the interview, parents consented to participate in the study. The project was briefly introduced, the procedure explained (including data handling, recording of the interview, and pseudonymization), informed consent obtained, and warm-up questions (e.g., asking about the child) asked. Prior to the interview, the parents were thanked and the incentive was provided, and room for questions was given.
